# Environmental scan of available guidelines for chlamydia and gonorrhea screening recommendations for non-pregnant adolescents/adults in developed countries

**DOI:** 10.14745/ccdr.v51i04a02

**Published:** 2025-04-03

**Authors:** Housne Begum, Dominique Basque, Michelle Haavaldsrud, Holly Sullivan, Stephan Gadient

**Affiliations:** 1 Public Health Agency of Canada, Ottawa, ON

**Keywords:** chlamydia, gonorrhea, screening, guidelines, public health

## Abstract

**Background:**

Over the past ten years, there has been a steady increase in the reported rates of gonorrhea and chlamydia in Canada, with gonorrhea rising by 171% and chlamydia by 26%.

**Objective:**

To collect and synthesize national and international chlamydia and gonorrhea screening guidelines to inform the revision of the current Public Health Agency of Canada (PHAC) recommendations.

**Methods:**

A scan of published chlamydia and gonorrhea screening guidelines of high-income countries was conducted. Guidelines were appraised using the Appraisal of Guidelines for Research & Evaluation II (AGREE II) and PROGRESS-Plus tools.

**Results:**

A total of 17 guidelines on chlamydia and gonorrhea screening published between 2015 and 2023 were included in this review. The overall score of the AGREE II methodological assessment ranged from a rating of three to seven out of seven points. Only one guideline fully met the considerations identified in the assessment tool. Most international organizations recommend universal screening for chlamydia, and a few organizations recommend opportunistic screening and targeted/risk-based screening. As for gonorrhea screening, organizations mostly recommend targeted/risk-based screening and a few organizations recommend universal screening. None of the international gonorrhea guidelines recommended opportunistic screening. The implementation of universal screening has been shown to have minimal negative impact on the individuals being screened, while increasing testing rates. Most guidelines recommend screening individuals <25 years of age, while only two organizations recommend screening individuals <30 years of age.

**Conclusion:**

The findings of this review will be used to inform the revision of the current PHAC recommendations on chlamydia and gonorrhea screening, which will be published in early 2025. International organizations recommend either universal or opportunistic screening. The majority of Canadian provinces and territories follow PHAC’s *Sexually Transmitted and Blood-Borne Infections: Guides for Health Professionals* and recommend universal screening for individuals <25 years of age.

## Introduction

In Canada, chlamydia and gonorrhea are the most common sexually transmitted infections (STIs) ((1)). If left untreated, these infections can lead to serious complications, such as chronic pelvic pain, pelvic inflammatory disease, infertility, ectopic pregnancy, epididymo-orchitis and reactive arthritis. Complications can be particularly severe in women ((2)). In Canada, rates of chlamydia and gonorrhea have increased steadily over the last decade; rates of chlamydia have increased by 26% and rates of gonorrhea by 171% ((1)). There was an exception to this trend during the COVID-19 pandemic ((3)), as demand and access to services related to STI decreased, likely impacting the rate of chlamydia and gonorrhea diagnoses in 2020 and 2021 ((1,4)). Notably, most STIs are asymptomatic, making it more difficult to detect and control cases, underrepresenting the rate increases. The purpose of chlamydia and gonorrhea screening is to detect asymptomatic infections before they cause further complications ((5)), to reduce transmission ((6,7)), and to maintain good sexual reproductive health ((8)). Screening programs should be implemented if the benefits exceed the harms, and the use of the resources is justifiable ((9)).

Many countries have assessed their chlamydia and gonorrhea screening programs to ensure their design, implementation and evaluation are based on the best available evidence. As an example, the National Chlamydia Screening Programme in England reports that the program’s aim is being changed to focus on preventing adverse consequences of untreated chlamydia infection, rather than aiming to reduce prevalence ((10)). The program has not found any clear evidence that widespread screening tests reduce chlamydia transmission, its prevalence and associated complications. Consequently, chlamydia screening will continue to be offered to females but will no longer be offered to males younger than 25 years old. Australia, on the other hand, recommends screening based on epidemiological findings. They recommend universal screening of males and females between the ages of 15 and 29 years for chlamydia and gonorrhea, as these infections are the most commonly notifiable infectious diseases among this age range ((11)).

Aside from age and sex, another critical component is the approach used for chlamydia and gonorrhea screening, as each approach has its own benefits and risks. Opportunistic screening is defined as screening offered opportunistically by clinicians in a variety of primary care settings during visits that may or may not be for sexual health-related concerns ((12)). This approach has the potential to normalize conversations about sexual health, sexual orientation and STIs between clinicians and patients, and thereby reduce stigma ((13)). On the other hand, risk-based screening has the benefit of targeting those who are most vulnerable to infection. However, it requires that individuals proactively self-identify factors that increase their risk level, possibly creating a barrier. Despite numerous and varying chlamydia and gonorrhea prevention and screening guidelines/programs, experts have reported that rates of chlamydia and gonorrhea continue to rise while testing rates remain low. Evidence-based interventions to screen and treat chlamydia and gonorrhea are needed to contain the STI epidemic and decrease the associated complications and the ensuing healthcare costs ((14)).

The National Advisory Committee on Sexually Transmitted and Blood-Borne Infections (NAC-STBBI) identified the review of its chlamydia and gonorrhea screening guidelines as one of its priorities. In 2021, the Canadian Task Force on Preventive Health Care (CTFPHC) updated their chlamydia and gonorrhea screening recommendations for adults and adolescents. This provided an opportunity for NAC-STBBI and the Public Health Agency of Canada (PHAC) to review and adopt, or adapt as appropriate, the new CTFPHC recommendation.

The Agency currently recommends annual universal chlamydia screening for individuals <25 years of age and targeted repeat screening based on risk factors in individuals ≥25 years of age ((15)). For gonorrhea, the recommendation is screening in asymptomatic sexually active individuals <25 years of age and other people with risk factors for STIs ((16)). The CTFPHC, on the hand, recommended opportunistic screening to be conducted for chlamydia and gonorrhea (annually) among sexually active individuals younger than 30 years of age who are not known to belong to a high-risk group for STI at primary care visits, using a self­ or clinician­ collected sample ((17)). This guideline differed from PHAC’s recommendations, as it relates to age, and the approach used for screening. To address this discrepancy, an environmental scan was conducted to collect and synthesize national and international chlamydia and gonorrhea screening guidelines and inform the revision of the current PHAC chlamydia and gonorrhea screening recommendations. The findings from the environmental scan and a systematic review of screening for chlamydia and gonorrhea were presented to a working group and used to develop recommendations, which will be published in early 2025.

## Methods

### Search strategy

To conduct this environmental scan, a search of previously published chlamydia and gonorrhea screening guidelines was conducted in March 2024. The US Institute of Medicine (IOM) defines clinical practice guidelines as statements that include recommendations intended to optimize patient care that are informed by a systematic review of evidence and an assessment of the benefits and harms of alternative care options ((18)). The search included Google, the websites of a few selected international organizations suggested by the working group (e.g., World Health Organization [WHO], United Kingdom Health Security Agency [UKHSA], United States Preventive Services Task Force [USPSTF], Centers for Disease Control and Prevention [CDC], Australasian Society for HIV, Viral Hepatitis and Sexual Health Medicine [ASHM], European Centre for Disease Prevention and Control [ECDC]) and provincial/territorial organizations. The websites of these organizations were chosen to explore/compare their guidelines for chlamydia and gonorrhea screening. The search was conducted using the key words, “guidelines,” “Neisseria gonorrhea,” “Neisseria gonorrhoeae,” “gonorrhea,” “gonorrhoea,” “chlamydia,” “chlamydia trachomatis,” “screening,” “testing” and “adults.” At the same time, a grey literature search was conducted that included searching sources identified by CTFPHC, as well as additional sources identified by the NAC-STBBI working group and secretariat. Sources searched included trial registries, conference abstracts, reports and chlamydia/gonorrhea screening guidelines from international and provincial/territorial public health organization websites.

### Inclusion and exclusion criteria

Guidelines on chlamydia and gonorrhea screening in non-pregnant adolescents and adults (>12 years of age) were included. These groups were the focus of this scan given that they are the target population of the guideline that is going to be updated. National guidelines were limited to high income countries, as defined by the World Bank Country List ((19)). This criterion limits the search to countries that have resources and infrastructure available to produce guidelines on STI screening. Furthermore, the healthcare landscape is more likely to be similar to the one in Canada, increasing the ability to make comparisons. Guidelines and documents that did not contain recommendations regarding who should be tested and screened, and/or recommendations regarding the timing of chlamydia or gonorrhea screening, were excluded, except for those of Canadian provinces and territories. Finally, in the case where a guideline or document had multiple versions available, the most recent version was included in the review. Guidelines not available in English or French were translated using an online translator.

### Data extraction and critical appraisal of the included guidelines

Summary tables of each guideline were created, including a summary of the recommendations, as well as findings from the Appraisal of Guidelines for Research and Evaluation II tool (AGREE II) ((20)) and PROGRESS-Plus ((21)). The AGREE II tool evaluates the methodological standards of clinical practice guideline development. Possible scores range from one to seven, with a higher score indicating a higher quality guideline. The tool has 23 items clustered into six domains (scope and purpose, stakeholder involvement, rigour of development, clarity of presentation, applicability and editorial independence). Finally, two overall scores are provided for guideline quality and recommendation for use.

Finally, PROGRESS-Plus factors (place of residence, race/ethnicity/culture/language, occupation, gender/sex, religion, education, socioeconomic status and social capital) were identified in the guidelines to assess the range of social determinants and factors that contribute to health equity ((21)). The retrieved guidelines were assessed by two members of the research team and data on the recommended age of screening and the screening approach used were extracted. In the case where the assessors did not agree, a third reviewer was included as a tie breaker. The findings were then synthesized and presented using tables.

## Results

### Environmental scan findings

A total of 17 organizations published guidelines on chlamydia and gonorrhea screening between 2015 and 2023. Of those 17, nine were international ((11, 22–32)) and eight were Canadian ((9,15,16,33–39)) (**Table 1**). The overall quality of the guidelines and the evidence used to develop the recommendations for the international and national organizations were assessed using the AGREE II tool. PROGRESS-Plus equity factors identified in the guidelines ((21)) were also identified, when available.

**Table 1:** International and federal/provincial/territorial guidelines/guidance on screening for gonorrhea and chlamydia

Organization, year, (country)	Infection type	Screening approach	Recommendation for <25 years or <30 years of age
**International guidelines**			
ASHM, 2021 (Australia) ((11))	CT & NG	Universal screening	<30 years
ECDC, 2015 (Europe) ((22))	CT	Opportunistic screening	<25 years
HAS, 2018 (France) ((23,24))	CT	Systematic (universal) and targeted opportunistic screening	<25 years
	NG	Targeted screening	No age recommendation
IUSTI, 2015 (Europe) ((25))	CT	Universal screening (under review)	<25 years
IUSTI, 2020 (Europe) ((26))	NG	Universal screening	<25 years
PH England, 2021 (England) ((27))	NG	Targeted screening	No age recommendation
UKHSA, 2022 (UK) ((28))	CT	Opportunistic screening	<25 years
CDC, 2024 (United States) ((29,30))	CT	Opportunistic screening	<25 years
	NG	Not specified	<25 years or >25 years who are at risk
USPSTF, 2015 (United States) ((31))	CT & NG	Universal screening and targeted risk screening	<25 years (women); >25 years who are at risk; no recommendations for men
The Dutch College of General Practitioners, 2023 (Netherlands) (32)	CT & NG	Targeted screening	<25 years
**Federal and provincial/territorial guidelines**			
CTFPHC ((9))	CT & NG	Opportunistic screening	<30 years
PHAC ((15,16))	CT	Universal and targeted screening	<25 years
	NG	Universal screening	<25 years
AHS ((33,34))	CT & NG	Universal screening	<25 years
HLBC ((35))	CT & NG	Universal screening and targeted screening	<25 years
Ontario ((36))	NG	Risk-based targeted screening	<25 years who are at risk
PEI ((37))	CT & NG	Universal screening	<25 years
Québec ((38))	CT & NG	Universal screening	<25 years
Yukon ((39))	CT & NG	Universal screening	<25 years

Abbreviations: AHS, Alberta Health Services; ASHM, Australasian Society for HIV, Viral Hepatitis and Sexual Health Medicine; CDC, Centers for Disease Control and Prevention; CT, chlamydia; CTFPHC, Canadian Task Force on Preventive Health Care; ECDC, European Centre for Disease Prevention and Control; HAS, Haute Autorité de la Santé; HLBC, HealthLink British Columbia; IUSTI, International Union Against Sexually Transmitted Infections; NG, gonorrhea; PEI, Prince Edward Island; PH England, Public Health England; PHAC, Public Health Agency of Canada; UKHSA, UK Health Security Agency; USPSTF, US Preventative Services Task Force

### International recommendations

Most international guidelines recommend a universal screening approach for chlamydia and gonorrhea infection in populations younger than 25 years of age ((22,23,25,26,28–32)), except for Australia ((11)) where the recommendation includes individuals up to 30 years of age, with no specificity on sex. An opportunistic approach to chlamydia screening is recommended by the CDC ((29)) and the ECDC ((22)) in males and females. As for gonorrhea screening, the CDC does not specify an approach to screening, but recommends screening for individuals less than 25 years old, or for those who are at higher risk for infection over the age of 25 years.

The UKHSA ((28)) and the *Haute autorité de la santé* (HAS) ((23)) recommend opportunistic chlamydia screening for individuals less than 25 years of age. These agencies recommend the same approach to screening for gonorrhea; however, there is no age recommendation ((28,24)). The USPSTF recommends universal and targeted screening for chlamydia and gonorrhea for sexually active females under the age of 25 years, or females that are 25 years and older and at risk of infection. They do not provide recommendations for males ((31)). Finally, the Netherlands does not test young people universally or opportunistically. Instead, they recommend assessing the risk in those less than 25 years of age who ask sex-related questions and omitting testing if the risk is negligible ((32)). Furthermore, the Netherlands guidelines do not provide recommendations for asymptomatic men and they also advise to no longer collect a urinalysis or material from the cervix or urethra if the woman is asymptomatic ((32)). Additional details on the recommendations for chlamydia and gonorrhea by organization are available in Table 1.

### National recommendations

The chlamydia and gonorrhea screening recommendations published by PHAC provide guidance to healthcare practitioners on screening of different groups. They recommend offering chlamydia screening to anyone who presents with risk factors for infection ((2,40)). Recommendations also include annual chlamydia screening for individuals under the age of 25 years, and for gay, bisexual, other men who have sex with men and transgender populations. They also recommend a targeted screening approach that is described as offering screening and repeat screening based on risk factors in those aged ≥25 years.

As for other published guidelines in Canada, screening for chlamydia in sexually active females up to the age of 25 years is recommended by all federal and provincial organizations ((15,16,33,35,37–39)), and up to the age of 30 years by the CTFPHC ((9)). In sexually active males, screening is recommended for individuals under 25 years of age by Canadian provinces ((33–38,41–44)) and territories ((39,45,46)) or under 30 years of age by the CTFPHC ((9)).

The Agency recommends screening for gonorrhea infections in asymptomatic sexually active people under 25 years, and anyone else with risk factors for sexually transmitted and blood-borne infection ((15,40)). All provincial and territorial guidelines include recommendations on gonorrhea screening. Universal screening for gonorrhea infection in females and males less than 25 years of age is recommended by several provinces and territories (PHAC ((15,16)), HealthLink BC ((35)), Alberta ((34)), Québec ((38)) and Yukon ((39))). Ontario recommends screening females and males less than 25 years of age only if they are at risk of contracting gonorrhea ((36)). The risk factors emphasized by Public Health Ontario include sexual contact with infected or symptomatic individuals, a history of STI, engagement in sex work, men who have unprotected sex with men, sexually active youth <25 years of age, street-involved youth, homelessness, having multiple partners, and travellers who had unprotected sex with a resident of an area with high gonorrhea rates and/or antibiotic-resistant gonorrhea ((36)). The CTFPHC recommends an opportunistic approach to gonorrhea screening in both females and males up to the age of 30 years ((9)). Most provinces and territories in Canada indicate that they have adapted their gonorrhea and chlamydia screening recommendations from the *Sexually Transmitted and Blood-Borne Infections: Guides for Health Professionals* ((47)).

### Guidelines appraisal

The guidelines did not fully meet all the considerations identified in the AGREE II tool (scope and purpose, stakeholder involvement, clarity of presentation, applicability, and editorial independence). Six out of 10 organizations had a methodology for their guideline development process. The USPSTF ((31)), the HAS of France ((23,24)), the UKHSA ((28)), the International Union Against Sexually Transmitted Infection (IUSTI) ((25,26)), the CDC ((29,30)) and the CTFPHC ((9)) used a systematic review to inform their own recommendation. The USPSTF ((31)), IUSTI ((25,26)), ASHM ((11)), the Dutch College of General Practitioners ((32)) and CTFPHC ((9)) applied a GRADE framework ((48)) to develop their recommendations. Other organizations used expert opinion, public comments, literature reviews, or adapted recommendations from other guidelines to inform the development of their recommendations. France HAS ((23,24)) and the UKHAS ((28)) included a cost-effectiveness component for the development of their recommendations. The AGREE II scaled domain percentages from highest to lowest among international recommendations were as follows: scope and purpose (81.3%), clarity of presentation (67.5%), stakeholder involvement (59.9%), editorial independence (46.4%), rigour of development (33.3%), and applicability (32.1%) ((11,22–32)). The overall score for the Canadian guidelines ranged from a rating of three to seven. The CTFPHC ((9)) is the only guideline that received a score of seven out of seven due to the quality of reporting, and for meeting the full criteria and considerations of the instrument. Health equity considerations, assessed using PROGRESS-PLUS, revealed that all guidelines published by international organizations considered women and men who have sex with men in the development of their recommendations. The same factors were considered in the development of all national guidelines, other than those produced by CTFPHC ((9)), where no considerations for the mentioned health equity factors were reported.

## Discussion

The purpose of screening for chlamydia and gonorrhea is to detect asymptomatic infection before it causes negative health outcomes, and to prevent further transmission in the general population. This environmental scan aimed to explore international and national guidelines on chlamydia and gonorrhea screening to inform the development of PHAC’s screening guidelines.

All international guidelines (other than the ECDC ((22))) have screening recommendations for both chlamydia and gonorrhea ((11,23–32)); however, some also include infection-specific recommendations ((22–30)). For instance, the CDC recommends chlamydia and gonorrhea screening for individuals <25 years old, but also recommend gonorrhea screening for individuals ≥25 years of age who are at risk ((29,30)). All international guidelines, other than ASHM, recommend screening individuals <25 years old for chlamydia and gonorrhea ((22–32)). Epidemiological findings on the age group with the highest incidence of chlamydia infections in Canada have been consistent ((1)). More specifically, from 2010 to 2021, the age group with the highest rates of chlamydia was 20–24 years, followed by the 25–29 year age group. Similar trends were observed between 2010 and 2018 for gonorrhea where rates were highest amongst the 20–24 year age group. However, as of 2019, rates became the highest in the 25–29 year age group ((1)). The assessment of the global burden of STI infections reports that the highest incidence of STIs is among individuals aged between 30 and 34 years ((49)), while in Europe, chlamydia and gonorrhea infection rates are highest among individuals aged 20–24 years, and 24–34 years, respectively ((50,51)). Therefore, the current recommended age in all of the reviewed guidelines for chlamydia and gonorrhea screening is only partially supported by international epidemiological reports. With that being said, adolescents and young adults are the most likely to engage in unprotected sex, making them at higher risk for sexually transmitted infection ((52–54)). This heightened risk among adolescents and young adults further supports the inclusion of a wider than a 20–24 year age criterion in public health screening programs.

The most common approach to chlamydia screening recommended by international organizations/governments is universal screening, followed by opportunistic screening, and targeted/risk-based screening. Conversely, in the case of gonorrhea screening, most guidelines recommend targeted/risk-based approaches to screening, then universal screening. No international gonorrhea guidelines recommend opportunistic screening. While universal screening has shown to increase testing rates in certain studies ((13,55,56)) it does not necessarily impact the infection positivity rates ((14,56)). The implementation of a universal screening approach has been shown to have minimal negative impact on the individuals being tested. In other words, universal screening as an approach may not necessarily decrease the number of observed cases of chlamydia and gonorrhea. These findings increasingly support the use of targeted-risk-based screening for gonorrhea infections, which could also be used for chlamydia screening.

Only half of the provinces and one territory have published screening guidelines; most align with the guidelines published by PHAC, making the provincial/territorial guidelines relatively consistent. Almost all provincial/territorial guidelines use the same recommendation for chlamydia and gonorrhea screening, with the exception of Ontario, which does not have a screening recommendation for chlamydia. Public Health Ontario refers healthcare professionals to PHAC for chlamydia screening recommendations. The guidelines recommend universal screening for individuals <25 years of age. Unlike most Canadian chlamydia and gonorrhea screening recommendations, many of the international guidelines have additional considerations, such as sex, risk assessments, and healthcare setting. For example, the USPSTF has only issued screening recommendations for females. These additional considerations are more in-line with a risk-based approach to screening, which could increase the number of infections detected, while also potentially increasing cost effectiveness ((57,58)).

The AGREE II tool assesses the quality of any guidelines, such as scope and purpose, stakeholder involvement, clarity of presentation, applicability, and editorial independence. The critical appraisal of the guidelines using this tool revealed that most did not meet all of the requirements, putting in question the quality of the recommendation. This can result in potential bias and issues relating to internal and external validity. The most common domains that were not met were rigour of development, stakeholder involvement, applicability, and editorial independence. The lack of rigour and systematic methods used to develop the guidelines included in this review could result in potential bias or a decrease in the reliability of the recommendations. The lack of stakeholder engagement, in other words not having the relevant professionals, viewpoints or target users involved in the creation of the recommendations, may have resulted in the omission of important viewpoints, which could negatively impact their applicability.

There are some gaps in the findings of this environmental scan that should be considered in using it to inform the development of chlamydia and gonorrhea screening guidelines. Firstly, the cost effectiveness of screening was not a part of this review. Future guidelines should consider cost, to ensure that the resources allocated to screening do not exceed the healthcare costs related to the current cases of chlamydia and gonorrhea infection in Canada. That being said, the expansion of screening programs has been shown to be cost-effective ((57,58)). Additionally, implementation instructions should be considered in the development of recommendations to increase their applicability. Ensuring that the implementation of the recommendations is feasible and could increase their use by healthcare professionals. In the context of the Canadian healthcare system, where federal governance does not supersede the provincial legislative and regulatory bodies, implementation pointers could be included in the guidelines. Finally, the engagement of patients, who would have a vested interest in screening guidelines, in their development could result in better accountability from the organizations and increase the applicability of the guideline.

## Limitations

There are some limitations to consider while interpreting the findings of this environmental scan. Firstly, the different published guidelines employed various screening approaches, such as universal screening, risk-based/targeted screening, or opportunistic screening. Unfortunately, not all organizations used the same definition, thus making comparison between organizations based on these definitions challenging. Secondly, in our review we may not have been able to retrieve all publicly available guidelines. For those we were able to access, not all made their appendices or supporting documents available, thus leading to lower scores when appraising them with the AGREE II tool.

## Conclusion

Although there is some variability between international and national guidelines, most recommendations focus on individuals in early adulthood and use a universal approach. It is critical to develop rigorous recommendations given the increasing rates of chlamydia and gonorrhea. The presented review highlights the need for guidelines that are developed carefully, with the aid of relevant stakeholders, considering applicability. The findings of this review will be used to inform the update of PHAC’s chlamydia and gonorrhea screening guidelines, which will support healthcare providers working within their respective provincial and territorial healthcare systems by producing recommendations that are clear, applicable and evidenced based. As a next step in the revision of the PHAC chlamydia and gonorrhea screening guidelines, a working group will consider this environment scan to update the recommendations in accordance with the findings of this scan and other evidence-based literature.

## Appendix

Supplemental material is available upon request to the author: sti.secretariat-its@phac-aspc.gc.ca
